# Supplementary material for: An evolutionary game analysis of algorithmic indirect copyright infringement from the perspective of collusion between UGC platforms and direct infringers
Source: PLoS One. 2024 May 15;19(5):e0292571. doi: 10.1371/journal.pone.0292571 (PMC11095730; doi:10.1371/journal.pone.0292571)
Supplement: Attachment — Submitted filename: PONE-D-23-30742_reviewer.docx [file pone.0292571.s002.docx]

**Title: Evolutionary game analysis of indirect copyright infringement by algorithms from the**

**the perspective of collusion between UGC platforms and infringers**

Review

\# Comment to the author:

The article focuses on the challenges and dynamics related to copyright infringement on user-generated content (UGC) platforms and the development of strategies and measures to address these issues, emphasising factors affecting infringement governance decisions and promoting stability and legality in the UGC ecosystem.

#Introduction:

The introduction reflects the discussion's content and meets the publication standard.

\# Model construction

The model description is understandable, using symbols to represent different variables. However, in #2.3 Model assumptions and benefits matrix:

Such as : 𝑉11 = 𝑦[−𝐶1 + 𝑎(𝐸1 − 𝑠𝐹 − 𝑠𝐺)] + (1 − 𝑦)(−𝐶1). The use of subscript would have been preferred for V11. i.e., $V_{11}$.

\# Impact of Infringement Compensation on the Outcome of Evolutionary Games after Successful Rights Defense:

i. Since simulated data are employed in describing the outcome of Evolutionary Games used in modelling infringement, more statistical output data need to be displayed
ii. Try to compute using the simulated data with the algorithm and show the outcome of at least 2 – 3 instances. This will enhance understanding of the propose algorithm.

#Figures - Labelling the graphs using English would be preferred for the reader to understand the pictorial view representing the outcome of the evolutionary game.

\# Countermeasures and recommendations

The recommendations reflect the importance of the algorithm proposed and demonstrated using a simulated dataset in the article. However, much is expected for the author to suggest future research based on a wealth of understanding gained in this domain while solving this problem.
