# Supplementary material for: An evolutionary game analysis of algorithmic indirect copyright infringement from the perspective of collusion between UGC platforms and direct infringers
Source: PLoS One. 2024 May 15;19(5):e0292571. doi: 10.1371/journal.pone.0292571 (PMC11095730; doi:10.1371/journal.pone.0292571)
Supplement: Attachment — Submitted filename: cover letter for response.docx [file pone.0292571.s003.docx]

Dear Editor and Reviewers:

On behalf of my co-authors, we thank you very much for giving us an opportunity to revise our manuscript, we appreciate editor and reviewers very much for their positive and constructive comments and suggestions on our manuscript entitled “An Evolutionary Game Analysis of Algorithmic Indirect Copyright Infringement from the Perspective of Collusion between UGC Platforms and direct Infringers”.

We have studied reviewer’s comments carefully and have made revision which marked in the paper. We have tried our best to revise our manuscript according to the comments.Pease find the revised version, which we would like to submit for your kind consideration.Revised portion are marked in yellow in the paper. The main corrections in the paper and the responds to the reviewer’s comments are as flowing:

Responds to the reviewers’s comments:

Reviewer#0:

Response to Additional Editor Comments:(*First, the study design is not clearly explained. It is unclear how the participants were selected and randomized, and the authors do not provide any information on the blinding of participants or investigators.* )

**Response:*We are grateful for the suggestion. To be more clear and in accordance with the reviewer concerns, we have added a brief description as follows: "Background information on evolutionary games is included in the text(line:111-118), explaining* why the use of evolutionary games was chosen to describe this type of infringement.The strategic choices of the participating subjects are also depicted in the form of a flowchart(line:160-169).”**

Reviewer#1:

Response to*#2.3 Model assumptions and benefits matrix:
Such as : 11 = [−1 + (1 − − )] + (1 − )(−1). The use of subscript would have been preferred for V11. i.e., V_11.*
**Response:We appreciate the heads-up and have made changes in the text(line183-188).**

Response to*#Impact of Infringement Compensation on the Outcome of Evolutionary Games after Successful Rights Defense:Since simulated data are employed in describing the outcome of Evolutionary Games used in modelling infringement, more statistical output data need to be displayed.Try to compute using the simulated data with the algorithm and show the outcome of at least 2 – 3 instances. This will enhance understanding of the propose algorithm.*

**Response:We have listened carefully to your comments. And we have added four cases of arithmetic evolution and added Figures 7 and 8 for easier understanding.(line:300-354).**

Response to*#Figures - Labelling the graphs using English would be preferred for the reader to understand the pictorial view representing the outcome of the evolutionary game.*

**Response:Your reminder is much appreciated and we have changed all the graphs in the text to English.**

Response to*#*Countermeasures and recommendations
The recommendations reflect the importance of the algorithm proposed and demonstrated using a simulated dataset in the article. However, much is expected for the author to suggest future research based on a wealth of understanding gained in this domain while solving this problem.

**Response:We greatly appreciate your suggestions and have included suggestions for future research at the end of the article.(line:448-463)**

Reviewer#2:

Response to*#2: 1- Modify reference 0 to reference 1 in the following statement: UGC (User Generated Content) platform refers to various forms of media and creative works created by users
through the Internet and its technology.0*

**Response:We appreciate your suggestions and have completed the changes in the text.**

Response to *2-Arranging the references mentioned in the introduction according to the year of publication (from oldest to newest) for the research review mentioned in this section*

**Response:Sorry, we didn't quite understand what you meant. But we have arranged the references from old to new and put them at the end of this letter. We hope it will meet your requirements.**

Response to*3-Adding a paragraph at the end of the introduction that explains the general structure of the research by clarifying each section.4- Standardize table content formats*

**Response:Thanks for the heads up, we've included a paragraph at the end of the introduction as an explanation(line:107-110). Harmonization of the formatting in the tables has also been completed.**

Response to *5- Section 2, in all its paragraphs, is devoid of reference to any reference. 6- Explaining the proposed algorithm in clear steps or through a flow chart that summarizes what has been explained*

**Response:*We are grateful for the suggestion. To be more clear and in accordance with the reviewer concerns, we have added a brief description as follows: "Background information on evolutionary games is included in the text(line:111-118), explaining* why the use of evolutionary games was chosen to describe this type of infringement.The strategic choices of the participating subjects are also depicted in the form of a flowchart(line:160-169).”**

Response to *7-The proposed future work has not been clarified to provide a future outlook for researchers in this field.*

**Response:We greatly appreciate your suggestions and have included suggestions for future research at the end of the article.(line:448-463)**

Response to *8-Standardize the format of references*

**Response:We really apologize for not standardizing the format of references and have made changes.**

Again, we would like to express our great appreciation to you and reviewers for comments on our paper. Looking forward to hearing from you.

Thank you and best regards.

Yours sincerely,

Jiangang Liu,Yuxuan Shen,Lanlan Zhou.

### References are listed from oldest to newest

[1] J.Maynard Smith, G.R.Price. The logic of Animal Conflict.Nature. 1973;246:15-18.
[2] Zheng Chengsi. Copyright Law [M]. Beijing:People's University of China Press. 1997;211-212 .
[3] Wang WB. Status and prospects of evolutionary game theory research. Statistics and Decision Making. 2009;03:158-161.
[4] Dellarocas C. Online reputation systems: How to design one that does what you need. MIT Sloan management review,. 2010;51(3): 33.
[5] Wang Liming. Interpretation of the Tort Liability Law of the People's Republic of China [M]. Beijing:China Law Press. 2010;158-159 .
[6] Wu Handong. On the Copyright Infringement Liability of Internet Service Providers. China Law Journal. 2011;02:38-47.
[7] Zhao Yuxiang, Fan Zhe, Zhu Qinghua. Conceptual analysis and research progress of user-generated content (UGC). Chinese Journal of Librarianship. 2012;38(05):68-81.
[8] Levina Arriaga M. Distinction and status production on user generated content platforms:Using Bourdieu’s theory of cultural production to understand social dynamics in online fields. Information Systems Research. 2014;25(3):468-488.
[9] Montgomery L, Priest E. Copyright and China's Digital Cultural Industries. Chapters. 2016.
[10] Gillespie T. Custodians of the Internet: Platforms, content moderation, and the hidden decisions that shape social media[M].Yale University Press. 2018.
[11] Myers West S. Censored, suspended, shadowbanned: User interpretations of content moderation on social media platforms. New Media&Society. 2018;20(11): 4366-4383.
[12] Jyh-An Lee. Tripartite perspective on the copyright-sharing economy in China. Computer Law & Security Review. 2019;35(4):434-452.
[13] Gupta D. Essays on the Management of Online Platforms:Bayesian Perspectives[D]. Virginian Tech. 2020.
[14] Gorwa R, Binns R, Katzenbach C. Algorithmic content moderation: Technical and political challenges in the automation of platform governance. Big Data&Society. 2020;7(1):2053951719897945
[15] Wang M, Lian S, Yin S, Dong H. A three-player game model for promoting the diffusion of green technology in manufacturing enterprises from the perspective of supply and demand. Mathematics. 2020;8(9):1585.
[16] Jin SY., Chen X. Evolutionary game analysis of digital transformation of accounting firms. China CPA. 2022;(05):23-29.
[17] Madio L, Quinn M. Content moderation and advertising in social media platforms.Available at SSRN. 2021;3551103.
[18] Liu Y, Yildirim T P, Zhang Z J. Social Media, Content Moderation, and Technology. arXiv preprint arXiv. 2101.04618,2021.
[19] Huang R, Xu Q, Li XW. A study on the evolutionary game of digital transformation of cultural industries. Theory and Practice of Finance and Economics. 2021;(02):125-133.
[20] Xiao HJ. Algorithmic responsibility: theoretical proof, panoramic portrait and governance paradigm. Management World. 2022;38(04):200-226.
[21] Tan J, Zhao XY. A study of technology strategies of innovation ecosystem players—based on evolutionary game and simulation of leading and following firms. Journal of Management Science. 2022;05:13-28.
[22] He Z, Zhang ZZ, Yang XH. An evolutionary game analysis of knowledge sharing incentives in cloud manufacturing innovation ecosystem. Chinese Management Science. 2022;07:77-87.
[23] Meng XY, Han SJ, Wu LL, Si SB, Cai ZQ. Analysis of epidemic vaccination strategies by node importance and evolutionary game on complex networks, Reliability Engineering & System Safety. 2022;219:108256.
[24] Quintais, J. P., de Gregorio, G., & Vieira Magalhães, J. How platforms govern users’ copyrightprotected content: Exploring the power of private ordering and its implications. Computer Law&Security Review. 2023;48:105792.
[25] Zhang P, Li C, Zhou M. Transaction transmission model for blockchain channels based on non-cooperative games. Sci. China Inf. Sci. 2023;66:112-105.
[26] Pei Y, Zhang MC, Zhou CX, Li AA. Dynamic evolutionary game-based modeling, analysis and performance enhancement of blockchain channels. IEEE/CAA J. Autom. Sinica. 2023;10(01):188-202.
