# Supplementary material for: An evolutionary game analysis of algorithmic indirect copyright infringement from the perspective of collusion between UGC platforms and direct infringers
Source: PLoS One. 2024 May 15;19(5):e0292571. doi: 10.1371/journal.pone.0292571 (PMC11095730; doi:10.1371/journal.pone.0292571)
Supplement: Attachment — Submitted filename: response to reviewers.docx [file pone.0292571.s004.docx]

Dear Editor and Reviewers:

On behalf of my co-authors, we thank you very much for giving us an opportunity to revise our manuscript, we appreciate editor and reviewers very much for their positive and constructive comments and suggestions on our manuscript entitled “An Evolutionary Game Analysis of Algorithmic Indirect Copyright Infringement from the Perspective of Collusion between UGC Platforms and direct Infringers”.

We have studied reviewer’s comments carefully and have made revision which marked in the paper. We have tried our best to revise our manuscript according to the comments.Pease find the revised version, which we would like to submit for your kind consideration.Revised portion are marked in yellow in the paper. The main corrections in the paper and the responds to the reviewer’s comments are as flowing:

Responds to Additional Editor Comments:

(1):The introduction provides a good, generalized background of the topic that quickly gives the reader an appreciation of the wide range of applications for this technology. This section helpfully explains the motivation for the research to current and potential funding agencies. However, to make the motivation clearer and to differentiate the paper some more from other applied papers, the author may wish to provide another sentence giving examples of some of the applications of this technology, along with appropriate references.

**Response:*We are grateful for the suggestion. To be more clear and in accordance with the reviewer concerns, we have added a brief description as follows: "References to scholars using evolutionary game methods to solve tort problems have been added(line:58-60);And papers on the many areas of evolutionary game use(line:122-125)*.”**

(2):There are several instances where assertions are made that are not substantiated with references. These have been noted in the appropriate sections of this paper. it would be better if you provide references to these assertions as this will make your work more concrete.

Author may wish to elaborate the problem formulation section and add some more details which will make the paper look more vital. I do not think any additional graphics are necessary.

**Response:We have listened carefully to your comment*s. About the assertion ,we have added References.(line:138-141,156-157).Regarding the assertion of replicating the dynamic part of the equation, we cite the proof of Friedman D's study(line:199-203).Regarding the problem formulation section, we have added detailed descriptions. The behavior of the participating subjects is portrayed in more detail.(line:132-136,150-152).***

(3):The author may also wish to give a more detailed discussion on blockchain and machine learning implementation.
The author may wish to mention why it is important to leverage blockchain to explain the motivation for his choice of specimens and accompany this with some references to other studies that demonstrate this importance.

**Response:We are very grateful for your reminder that brought to my attention the lack of a blockchain as well as a machine learning literature section.We have included references to blockchain and machine learning in the literature review section.*(line:79-85)***

Responds to Review Comments to the Author:

**Response:We have listened very carefully to your suggestions and have enriched the research in this paper by citing the references that you have recommended.These recommended references are located at the 15th, 20th, and 17th position of the citation.**

Again, we would like to express our great appreciation to you and reviewers for comments on our paper. Looking forward to hearing from you.

Thank you and best regards.

Yours sincerely,

Jiangang Liu,Yuxuan Shen,Lanlan Zhou.
